# Changes in immune activation in the T Cell compartments of HIV HCV coinfected patients during PEG IFN RBV treatment

**DOI:** 10.1186/1742-4690-9-S1-P56

**Published:** 2012-05-25

**Authors:** Amélie Menard, Corinne Brunet, Véronique Obry, Patrick Dukan, Sylvie Brégigeon, Olivia Faucher, Estelle Balducci, Anne-Suzel Ritleng, Françoise Dignat-George, Isabelle Poizot Martin

**Affiliations:** 1 Haematology-Aids Unit Hôpital Sainte-Marguerite, Marseille, France

## 

Chronic activation of CD8 T-cell compartment is critical during HCV-HIV-1 coinfection. The objective of this study was to evaluate the impact of pegylated-interferon (PEG-IFN) in combination with ribavirin (RBV) on immune activation in HCV-HIV-coinfected patients.

T cell phenotype (CD8+CD38+,CD8+DR+) was quantified using flow cytometry analysis,. Measurements were performed at day one of treatment (Baseline, BL), then at week (W)12,W24,W48 and W24 post-treatment. HVC viral load was measured using a PCR (COBAS TaqMan 48; Roche), exhibiting a limit of detection at12 IU/ml. Statistical analysis was performed with SPSS 17.0.

11 pts (64% of males; median age 47.4 [45.1-51]) with a median follow up for HCV infection of 14.7y [11.7; 19.1]) were evaluated. All were treated for HIV infection (PI- based regimen: 63.6%) with an HIV-VL < 40copies/ml. Median CD4 and CD8 T cells count at BL was 886/mm3 [671; 1008] and 825/mm3 [530; 1843], respectively. HCV genotype was 1 for 63.6%, 3 in 27.3% and 4 in one pt. HCV VL at BL was 5.9 [4.6; 6.7] (log UI/ml). Up to now, 2 pts stopped HCV treatment at W2 and W4, 9 pts have reached W24, and 5 of them are between W24 and W48. The results at W12 and W24 are presented in the table. We observed a significant decrease of the number of circulating total lymphocytes and CD4T cells in absolute value (p=0.008), but a significant increase in the percentage of CD4+ T-cells and a significant decrease in the percentage of CD8+ T cells at W24. HCV VL was negative for all of them.

These preliminary results show that the immune system hyperactivation driving by HCV disease can be reduced with a control of HCV replication. However, we observed a discrepancy in the evolution of CD8+CD38+ and CD8+DR+ expression at W12 which remains at W24.These results have to be confirmed with the next measurement performed at W48 and W24 post treatment.

**Table 1:** 

	BL	W12	p	W24	p
CD4+ T Cell (%)	27.0 [24.5; 37.1]	37.5 [26.8; 46.0]	0.008	43.0 [30.5; 47.3]	**0.011**
CD8+ T Cell (%)	46.0 [30.4; 51.0]	43.2 [25.9; 49.7]	0.110	37.4 [26.5; 46.9]	**0.028**
DR+CD8+ T Cell (%)	11.0 [6.4; 19.5]	5.2 [2.8; 9.9]	0.017	2.4 [1.4; 7.4]	**0.008**
CD38+CD8+ T Cell (%)	14.7 [6.8; 21.5]	43.2 [28.9; 48.9]	0.008	36.2 [15.7; 47.1]	**0.015**

